# Women, culture and the HIV epidemic in MENA

**DOI:** 10.7448/IAS.17.1.19074

**Published:** 2014-03-08

**Authors:** Renu Chahil-Graf, Navid Madani

**Affiliations:** 1 Former Director, UNAIDS Middle-East and North Africa, Regional Support Team, Cairo, Egypt; 2 Research Scientist, Department of Cancer Immunology and AIDS, Dana-Farber Cancer Institute, Department of Global Health and Social Medicine, Harvard Medical School, Boston, MA, USA

Historical analyses of the cultural impact of infectious diseases are quite popular, as demonstrated by the success of Jared Diamond's book *Guns, Germs, and Steel* [1]. Assessing the impact of culture on epidemics, however, should not be thought of as a mere exercise but is of absolute importance to understand how cultural customs and beliefs shape a society's response to a disease.

Nowhere is this clearer than in the case of the AIDS epidemic, wherein cultural constraints, such as low status of women, have contributed to increased HIV transmission in the Middle East and North Africa (MENA). As a sexually transmitted disease, AIDS incites moral reactions that complicate the fight against the deadly HIV pathogen. The early response to AIDS in many countries – developing and developed – was deeply impacted by homophobia. Countries need to identify and address cultural factors that could endanger vulnerable populations. In addition, rather than being considered an “impediment,” can the local cultural context be “leveraged” to find workable solutions?

In MENA, the incidence of HIV infection is increasing [2]; in fact, this region now exhibits one of the fastest increases in HIV infection rates in the world. Because behaviours associated with HIV transmission are culturally prohibited or illegal, such as drug use, commercial sex and sex between men, it becomes even more difficult to address the issue. While the incidence of infections have been increasing in both male and female populations, in some countries, the incidence of infection among women between the years 2001 and 2012 have increased exponentially – for example, from an estimate of 4,400 to 11,000 women infected in Morocco, and from 1,300 to 7,700 in Yemen [3].

There are many factors contributing to this increase. A recent paper points to sparse epidemiological data for the region [2], another to inadequate understanding of the populations at risk [4]. Among these causes, though, lies lack of attention to social and cultural structures; indeed, research points to insufficient investigation into cultural norms that impact the HIV epidemic [5].

At the outset of the global HIV epidemic, the major route of transmission was homosexual contact, with the majority of infections occurring in males. By the early 1990s, however, HIV infection rates in the United States were increasing faster among young women than their male peers [6]. Women account for 44% of adults living with HIV in the MENA region, their risk primarily a consequence of the behaviour of their partners. This has been recurring as the AIDS epidemic “matured” in different countries [7]. Recent data, though limited, indicate that the HIV epidemic in the MENA region is following this trend [8, 9].

The burden of the HIV epidemic is increasingly impacting women in a region where they are marginalized and lack comprehensive sexual and reproductive health services, including HIV testing. Women who engage in sex work have one of the highest rates of HIV in the MENA region [10]. However, because of economic decline, war and civil unrest in the region in recent years, the vulnerability of women in general has increased, for instance through transactional sex, infection through partners and the very low levels of treatment. The vulnerability of women and girls to HIV is a reflection of deeper inequalities many of which are embedded in law, culture and traditional practices. For example, child marriage is still common in the poorest countries such as Yemen, Sudan and Somalia. Female genital mutilation is common practice in at least five countries of the region [7].

Cultures are deeply engrained and are not easy, nor quick, to change. However, HIV will not wait. Working within existing cultural contexts is essential for stopping the spread of the HIV epidemic in MENA.

HIV has been addressed in MENA by working in novel and innovative ways. One such example is through the regional association MENA-Rosa, launched in 2010 by a group of women living with HIV who established this network, with the support of UNAIDS, to be able to articulate and advocate for their needs and define solutions [10]. Numerous countries in the region have piloted innovative approaches to reaching women. For example, in Iran, positive clubs and drop-in centres for people living with HIV provide peer education, care, treatment, and psychosocial support. These facilities also offer assistance to HIV-positive women in developing new skills to enhance their economic status and to enter the workforce. In Algeria, the NGO El Hayet coordinates projects to ensure the socio-economic re-entry of women affected by HIV into the workforce. Others work towards preventing the transmission of HIV from mothers to children. In Morocco, a four-year national plan for elimination of mother-to-child transmission of HIV was initiated in 2012 with the innovative feature of engaging private health entities to provide HIV testing and awareness programmes for pregnant women. In addition, another powerful approach in the region is the work of female religious leaders and Imams who have been trained to communicate prevention and awareness messages to women in religious institutions. These are but a few illustrations of efforts to leverage stronger partnerships. Can these efforts be expanded throughout the larger community? Can men be brought into the conversation?

To achieve this, policy actions such as female-sensitive health care, and social equality – in education, legal rights, employment opportunities – must move forward with urgency. However, it takes time to change structures and cultures, and HIV spreads rapidly. Thus, concurrently, women-led and conceived community actions and initiatives must be supported and funded. Change must be brought about by working within the existing cultural context. Engaging with women, in partnership with responsive men, will not only help protect women with HIV but also stop the AIDS epidemic and reverse its spread in the MENA region. The mantra in this millennium is to work towards an AIDS-free generation. An AIDS-free generation starts with empowering and engaging women in every culture, religion and setting.
